# *Epimedium koreanum* Extract and Its Constituent Icariin Improve Motor Dysfunction in Spinal Cord Injury

**DOI:** 10.1155/2012/731208

**Published:** 2012-08-23

**Authors:** Chihiro Tohda, Aiko Nagata

**Affiliations:** Division of Neuromedical Science, Institute of Natural Medicine, University of Toyama, Toyama 930-0194, Japan

## Abstract

Although cell transplantation strategies for spinal cord injury (SCI) using sources such as iPS cells and neural stem cells are focused as expectative therapies for SCI, the possibility of medication as more accessible and practical way should not be given up. We, therefore, aimed to develop medical sources for SCI. In this paper, we evaluated effects of a famous tonic herb, *Epimedium koreanum*, on motor dysfunction in spinal cord injury (SCI). The spinal cord was injured by contusion after laminectomy at T10 level. Oral administration of the methanol extract of *E. koreanum* significantly enhanced hindlimb function in SCI mice by short period treatment (for initial 3 days) and chronic treatment (21 days), although chronic treatment recovered the function more potently. Since it is well known that icariin is the major constituent in *E. koreanum*, icariin was administered orally to SCI mice for initial 3 days. Motor dysfunction was ameliorated by icariin treatment similarly to the methanol extract of *E. koreanum*. This paper is the first report to indicate *E. koreanum* is effective for recovery of motor function in SCI, and at least icariin is an active constituent.

## 1. Introduction

Approximately 2,500,000 people worldwide suffer from spinal-cord-injury- (SCI-) induced chronic paralysis (Risk Hansen Spinal Cord Injury Registry; http://rickhansenregistry.org/), for which no effective drug therapy currently exists. SCI causes serious locomotor dysfunction due to the disruption of the descending motor and ascending sensory tracts at the lesion site.

Methylprednisolone is used in the standard treatment of acute SCI at present, which is based on the results of clinical multicenter studies such as National Acute Spinal Cord Injury Studies II and III and a Japanese trial [1]. However, the effects of methylprednisolone on functional recovery have been questioned [2, 3]. Although cell transplantation strategies for spinal cord injury (SCI) using sources such as iPS cells and neural stem cells are focused as expectative therapies for SCI, the possibility of medication as more accessible and practical way should not be given up.

During the subacute phase after SCI, locomotion spontaneously, but slightly, improves [4]. At this phase, the growth of axons and the increase in the number of Schwann cells occur in the injured site [5]. Therefore, promoting the healing process after SCI must be a key strategy in treating SCI. We previously found axonal growth effects of several tonic herbal medicines and those derived steroidal saponins [6–9]. Among them, withanoside IV, which is constituent in an ayurvedic tonic medicine, ashwagandha (root of *Withania somnifera* Dunal), [7] which was evaluated for the improvement of motor dysfunction using SCI mice. Oral administration of withanoside IV to SCI mice recovered hindlimb dysfunction, enhanced axonal regrowth, and increased peripheral myelin levels in the injured spinal cord [10]. In addition, we also found the effect of other steroidal sapogenin on SCI injury and its molecular mechanism (submitted). We focus on a famous tonic herb, Epimedii Herba. It is well known that flavonoids such as icariin are rich in Epimedii Herba, especially in *Epimedium koreanum* (Mdideanetph (2010, http://www.mdidea.net/products/herbextract/icariin/data10.html). In this paper, we aim to investigate effects of the extract of *E. koreanum* on SCI injury.

## 2. Materials and Methods

All experiments were performed in accordance with the Guidelines for the Care and Use of Laboratory Animals at the Sugitani Campus of the University of Toyama and the NIH Guidelines for the Care and Use of Laboratory Animals. The Committee for Animal Care and Use at the Sugitani Campus of the University of Toyama approved each of the study protocols. All efforts were made to minimize the number of animals used.

### 2.1. Preparation of Extract

The methanol extracts of *E. koreanum* Nakai (Tochimoto Tenkaido, Osaka, Japan) were prepared as follows. Fifty grams of grained aerial part of *E. koreanum* were placed in methanol (750 mL) for 3 h at 75°C. The supernatant was evaporated to obtain the methanol extracts at 35–40°C. Yield of the extract was 13.9%. The extracts were dissolved in dimethyl sulfoxide (DMSO) for experiments at 1,000 times concentration of the final concentration.

### 2.2. Animals and SCI Model Experiments

**Figure 1:**
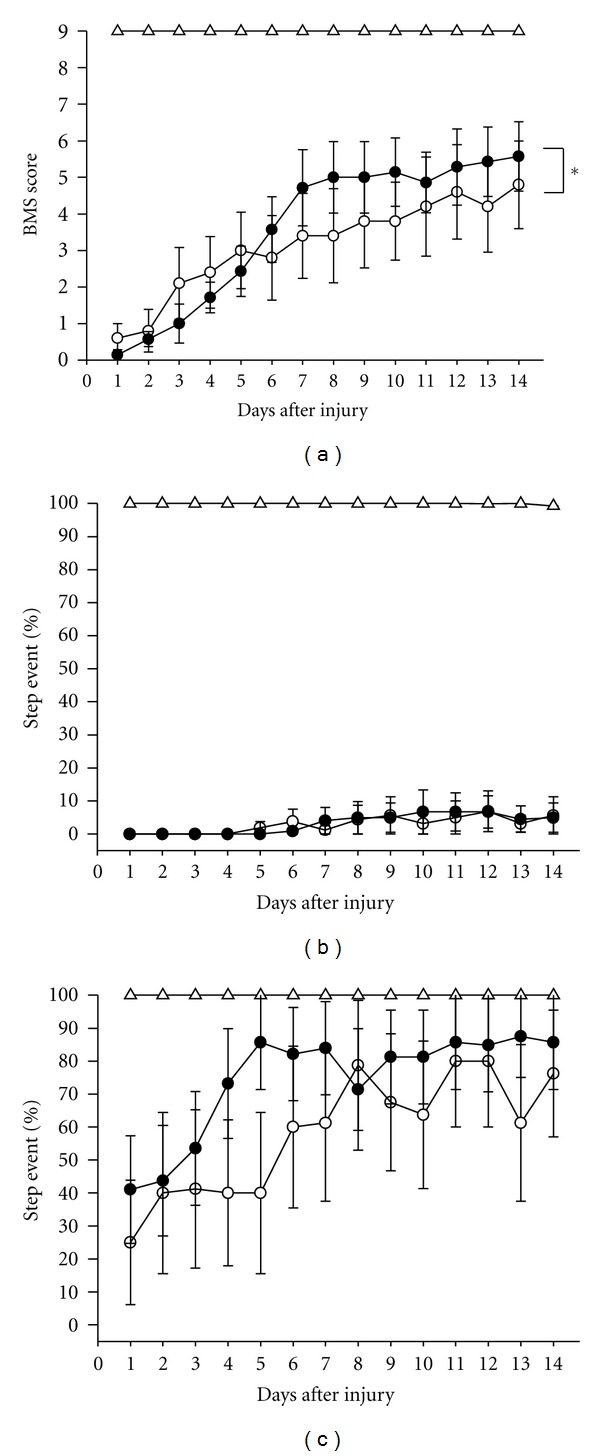
Effects of short treatment of the methanol extract of *E. koreanum* on hindlimb function in SCI mice. BMS score (a), the percentages of step event (b), and success rate (c) in ladder test were measured. SCI mice were administered the methanol extract of *E. koreanum* (100 mg/kg, p.o., closed circles, 7 mice, 14 hindlimbs, *n* = 14 (a) and (b), *n* = 7 (c)), or vehicle solution (open circles, 5 mice, 10 hindlimbs, *n* = 10 (a) and (b), and *n* = 5 (c)) for initial 3 days. Sham operated mice were also tested (open triangles, 7 mice, 14 hindlimbs, *n* = 14 (a) and (b), and *n* = 7 (c)). *Repeated measures two-way ANOVA followed by the *post hoc* Bonferroni test, *P* < 0.05.

**Figure 2:**
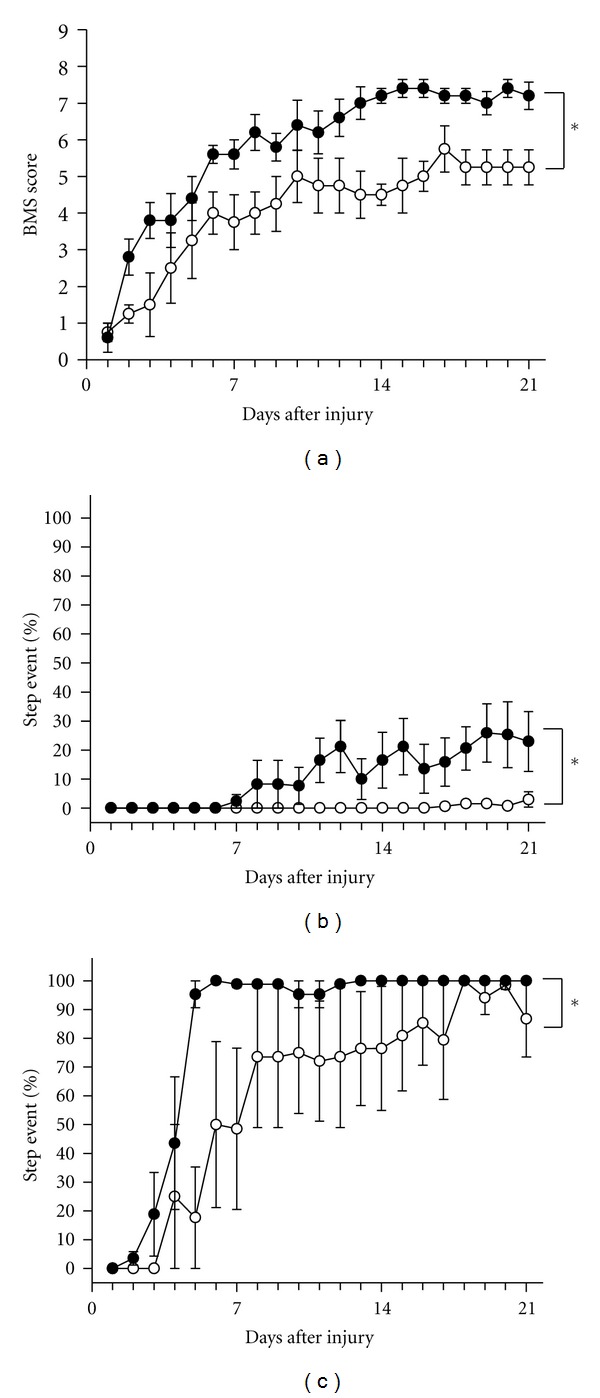
Effects of chronic treatment of the methanol extract of *E. koreanum* on hindlimb function in SCI mice. BMS score (a), the percentages of step event (b), and success rate (c) in ladder test were measured. SCI mice were administered the methanol extract of *E. koreanum* (100 mg/kg, p.o., closed circles, 5 mice, 10 hindlimbs, *n* = 10 (a) and (b), *n* = 5 (c)) or vehicle solution (open circles, 4 mice, 8 hindlimbs, *n* = 8 (a) and (b), *n* = 4 (c)) for 21 days. *Repeated measures two-way ANOVA followed by the *post hoc* Bonferroni test, *P* < 0.05.

**Figure 3:**
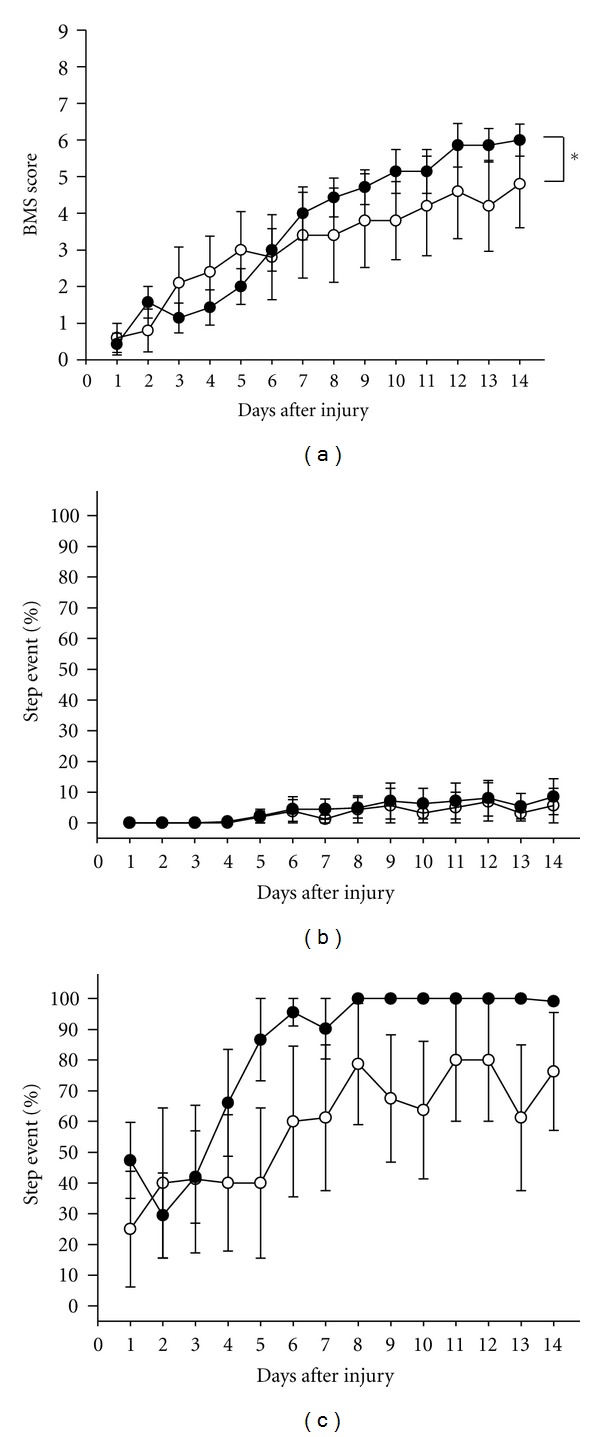
Effects of short treatment of icariin on hindlimb function in SCI mice. BMS score (a), the percentages of step event (b), and success rate (c) in ladder test were measured. SCI mice were administered icariin (50 *μ*mol/kg, p.o., closed circles, 7 mice, 14 hindlimbs, *n* = 14 ((a) and (b)), and *n* = 7 (c)) or vehicle solution (open circles, 5 mice, 10 hindlimbs, *n* = 10 ((a) and (b)), and *n* = 5 (c)) for initial 3 days. *Repeated measures two-way ANOVA followed by the *post hoc* Bonferroni test, *P* < 0.05.

Seven-week-old male ddY mice (SLC) were used for the SCI experiments. The mice were housed with ad libitum access to food and water and were maintained under constant environmental conditions (22 ± 2°C, 50 ± 5% humidity and 12-hr light: 12-hr dark cycle starting at 07:00). The surgical operations to produce SCI were performed as described previously [11] with slight modifications. After laminectomy at T10 level, contusion injuries were produced by three times dropping a 2.5-g weight from a height of 3 cm onto the exposed dura mater of the lumbar spinal cord L1 level using a stereotaxic instrument (Narishige, Tokyo, Japan). One hour after surgery, the SCI mice were randomly divided into the vehicle-treated and drug-treated groups, and application of the drug was initiated. The methanol extract of Epimedii Herba at 100 mg/kg or a vehicle solution (0.1% DMSO in saline) was administered orally once daily to the animals for 3 days (Figure 1) or 21 days (Figure 2). Icariin (50 *μ*mol/kg) and the vehicle solution (0.1% DMSO in saline) were administered orally once daily to mice for 3 days (Figure 3). The dose of methanol extract of *E. koreanum *(100 mg/kg, p.o.) used here was based on other *in vivo* study [12]. Icariin content in the methanol extract used in this study was 1.933%, suggesting 2.85 *μ*mol/kg icariin should be contained in the 100 mg/kg extract. Icariin (purity 99.0%) used in this study was purchased from Wako (Osaka, Japan).

### 2.3. Behavioral Evaluation

For behavioral scoring, the mice were individually placed in an open field (36 cm × 30 cm × 17 cm) and observed for 5 min. Open-field locomotion was evaluated using the 0–9-point BMS-locomotion scale for 21 days (Figure 2) or 14 days (Figures 1 and 3). In horizontal ladder test, the ladder was 72-cm long and 5-cm wide, and consisted of 5-mm diameter plastic rungs spaced regularly (every 4.5-cm interval). At each testing period, walking ability on the 17 rungs across the ladder were recorded, and the percentages of step event and success rate were determined. The step event shows plantar steps on rungs without slipping or dragging. The success rate shows the percentage of the number of rungs that the mouse can reach without dropping from the ladder regardless walking pattern of hindpaws. Animal numbers of extract-, vehicle-, and sham-treated mice were 7, 5, and 7, respectively, in Figure 1. Animal numbers of extract- and vehicle-treated mice were 5 and 4, respectively in Figure 2. Animal numbers of icariin- and vehicle-treated mice were 7 and 5, respectively, in Figure 3.

### 2.4. Statistical Analysis

Statistical comparisons were performed using a repeated measure two-way ANOVA followed by the *post hoc* Bonferroni test. The SigmaStat 3.5 (SYSTAT) was used for the statistical analyses. *P* values < 0.05 were considered significant. The mean values of the data are presented along with the SE.

## 3. Results

Motor function of hindlimb was measured by BMS score and a horizontal ladder test. Two hindlimbs were separately scored in both tests. BMS evaluates ankle movement and walking stability [13]. The horizontal ladder test evaluates walking ability and skill. In our SCI model, the BMS score spontaneously and gradually increases and reaches the plateau at 14 days after the injury in an injured control group. The methanol extract of *E. koreanum* (100 mg/kg, p.o.) was administered to the SCI mice for initial 3 days, and BMS score and horizontal ladder test score were measured for 14 days. BMS scores for 14 days in the extract-treated group significantly higher than that in injured control group (Figure 1(a)). Repeated measures two-way ANOVA revealed significant time × drug interaction (*F*(13, 285) = 1.824, *P* = 0.039). Sham operation group showed 9 points during the experimental period. In the horizontal ladder test, step event percentages in control and extract-treated groups were very low at similar degrees (Figure 1(b)). However, the success rate in extract-treated group tended to be higher than that in control (Figure 1(c)).

 Next, the methanol extract of *E. koreanum* (100 mg/kg, p.o.) was administered to the SCI mice for 21 days. BMS scores for 21 days in the extract-treated group significantly higher than that in injured control group (Figure 2(a)). Repeated measures two-way ANOVA revealed significant time × drug interaction (*F*(20, 320) = 1.93, *P* = 0.0104). The scores at endpoint were 7.2 and 5.25 in extract-treated group and control, respectively. The point 7 indicates parallel plantar stepping with coordination. The point 5 indicates rotated plantar stepping without coordination. In horizontal ladder test, the step event percentages in extract-treated groups significantly higher than that in injured control (Figure 2(b)). Repeated measures two-way ANOVA revealed significant time × drug interaction (*F*(20, 320) = 2.48, *P* = 0.0005). The success rate was also significantly increased by extract treatment compared with injured control (Figure 2(c)). Repeated measures two-way ANOVA revealed significant time × drug interaction (*F*(20, 140) = 2.28, *P* = 0.0029).

Since, flavonol glycoside icariin is a major constituent in *E. koreanum*, we administered icariin (50 *μ*mol/kg, p.o.) to the SCI mice for 3 days, and BMS score and horizontal ladder test score were measured for 14 days. BMS scores for 14 days in the icariin-treated group were significantly higher than that in control group (Figure 3(a)). Repeated measures two-way ANOVA revealed significant time × drug interaction (*F*(13, 285) = 4.108, *P* < 0.0001). The score at endpoint was 6.0 in icariin-treated group. In horizontal ladder test, the step event percentages in icariin-treated group was not different from that in injured control (Figure 3(b)). However, the success rate in icariin-treated group tended to be higher than that in control (Figure 3(c)). The icariin content in the methanol extract of* E. koreanum *used in this study was determined as 1.933% by quantitative HPLC. This content is in the range reported value (1.55–3.69%) (Mdideanetph (2010, http://www.mdidea.net/products/herbextract/icariin/data10.html). In the methanol extract of *E. koreanum* used in Figures 1 and 2 (100 mg/kg), approximately 2.85 *μ*mol/kg of icariin should be contained.

## 4. Discussion

*E. koreanum* is distributed in Korea, and traditionally used for impotence, spermatorrhoea and forgetfulness in Korea [13]. The main active constituent in *E. koreanum* and icariin has a variety of bioactivities. Antitumor [14], antioxidant effect [15], cardiovascular function [16], antidepressant [17], regulation of immune system [18], and anti-Alzheimer's disease [19]. This paper is the first to report ameliorative effects of the methanol extract of *E. koreanum *and its constituent icariin on SCI.

It is quite interesting that even short treatment with the extract and icariin for only initial 3 days (Figures 1 and 3) showed significant effects on hindlimb function although the potency was less than that in long treatment. In acute phase and early subacute phase in SCI, cell death from direct insult and apoptosis markedly occur [20]. These may suggest that neuroprotective effect is important for functional recovery especially in early phase in SCI. Several reports indicate neuroprotective effect of icariin on oxidative stress-induced damages [21, 22]. The mechanism of icariin was mainly supposed as inhibition of p38 MAPK [23–25]. The p38 mitogen-activated protein kinase (MAPK) is one of the key enzymes in apoptosis induction pathways. Continuous intrathecal infusion of SB203580, a selective inhibitor of p38-MAPK, recovered the frequency of standing 2-3 weeks after the spinal cord injury [25]. Inhibition of p38 effectively reduced iNOS mRNA expression and rescued neurons from apoptosis and death in the area adjacent to the lesion epicenter in SCI [26]. As a next step, we need to investigate that neuroprotective effect of icariin in the lesion center and activity change of p38 MAPK by icariin.

Treatment with the methanol extract of *E. koreanum* for 21 days provided effect on hindlimb function more potently than treatment for 3 days (Figure 2). This data suggests that continuous treatment with the *E. koreanum* extract may protect the ongoing injury progress during the subacute phase. Although spontaneous recovery in SCI was shown in BMS scores, the stepping event in the ladder test is the more severe index for evaluation and was nearly 0% in injured control during observations. Because plantar stepping on rungs needs hindlimb ability above point 6 in BMS. In contrast, chronic treatment with the methanol extract for 21 days markedly increased the step event from 8 days after injury, showing that mice were able to put their weight on hindpaws. These results indicate that *E. koreanum* enhances functional recovery in SCI. Although icariin treatment was done only for 3 days in this study, more potent ameliorative effect is expected when icariin is administered for a long time.

## 5. Conclusions

*E. koreanum* is effective for recovery of motor function in SCI, and at least icariin is an active constituent. Although effects of the methanol extract of* E. koreanum *are observed even by short-term treatment, chronic treatment enhances hindlimb function more potently. The effects of *E. koreanum* and icariin for SCI may provide the useful information for developing new therapeutic medicines for SCI.

## References

[1] Fehlings MG, Baptiste DC (2005). Current status of clinical trials for acute spinal cord injury. *Injury*.

[2] Kronvall E, Sayer FT, Nilsson OG (2005). More and more questions about the use of methylprednisolone for the treatment of acute spinal cord injury. *Lakartidningen*.

[3] Weaver LC, Gris D, Saville LR (2005). Methylprednisolone causes minimal improvement after spinal cord injury in rats, contrasting with benefits of an anti-integrin treatment. *Journal of Neurotrauma*.

[4] Okada S, Nakamura M, Katoh H (2006). Conditional ablation of Stat3 or Socs3 discloses a dual role for reactive astrocytes after spinal cord injury. *Nature Medicine*.

[5] Pearse DD, Pereira FC, Marcillo AE (2004). cAMP and Schwann cells promote axonal growth and functional recovery after spinal cord injury. *Nature Medicine*.

[6] Kuboyama T, Tohda C, Komatsu K (2005). Neuritic regeneration and synaptic reconstruction induced by withanolide A. *British Journal of Pharmacology*.

[7] Kuboyama T, Tohda C, Komatsu K (2006). Withanoside IV and its active metabolite, sominone, attenuate A*β*(25–35)-induced neurodegeneration. *European Journal of Neuroscience*.

[8] Tohda C, Matsumoto N, Zou K, Meselhy MR, Komatsu K (2004). A*β*(25–35)-induced memory impairment, axonal atrophy, and synaptic loss are ameliorated by MI, a metabolite of protopanaxadiol-type saponins. *Neuropsychopharmacology*.

[9] Tohda C, Joyashiki E (2009). Sominone enhances neurite outgrowth and spatial memory mediated by the neurotrophic factor receptor, RET. *British Journal of Pharmacology*.

[10] Nakayama N, Tohda C (2007). Withanoside IV improves hindlimb function by facilitating axonal growth and increase in peripheral nervous system myelin level after spinal cord injury. *Neuroscience Research*.

[11] Krenz NR, Weaver LC (2000). Nerve growth factor in glia and inflammatory cells of the injured rat spinal cord. *Journal of Neurochemistry*.

[12] Kang HK, Choi Y-H, Kwon H Estrogenic/antiestrogenic activities of a Epimedium koreanum extract and its major components: in vitro and in vivo studies. *Food and Chemical Toxicology*.

[13] Basso DM, Fisher LC, Anderson AJ, Jakeman LB, McTigue DM, Popovich PG (2006). Basso mouse scale for locomotion detects differences in recovery after spinal cord injury in five common mouse strains. *Journal of Neurotrauma*.

[14] Li CL, Zhang L, Gu HT (2007). The in vivo anti-tumor effects of Icariin and its mechanisms. *Chinese Journal of Cancer Biotherapy*.

[15] Li L, Wu Q, Zhou QX, Shi JS (2005). Protective effect of icariin against mitochondrial damage induced by oxygen free radical in rat cerebral cells. *Chinese Journal of Pharmacology and Toxicology*.

[16] Pan ZW, Wang QJ, Xu J, Du BS, Kong LY (2007). Effects of icariin on the isolated hearts and hemorheology of rats. *Journal of China Pharmaceutical University*.

[17] Pan Y, Kong LD, Xia X, Zhang WY, Xia ZH, Jiang FX (2005). Antidepressant-like effect of icariin and its possible mechanism in mice. *Pharmacology Biochemistry and Behavior*.

[18] Xu CQ, Liu BJ, Wu JF (2010). Icariin attenuates LPS-induced acute inflammatory responses: involvement of PI3K/Akt and NF*κ*B signaling pathway. *European Journal of Pharmacology*.

[19] Urano T, Tohda C (2010). Icariin improves memory impairment in Alzheimer’s disease model mice (5xFAD) and attenuates amyloid *β*-induced neurite atrophy. *Phytotherapy Research*.

[20] Oyinbo CA (2011). Secondary injury mechanisms in traumatic spinal cord injury: a nugget of this multiply cascade. *Acta Neurobiologiae Experimentalis*.

[21] He XL, Zhou WQ, Bi MG, Du GH (2010). Neuroprotective effects of icariin on memory impairment and neurochemical deficits in senescence-accelerated mouse prone 8 (SAMP8) mice. *Brain Research*.

[22] Wang L, Zhang L, Chen ZB, Wu JY, Zhang X, Xu Y (2009). Icariin enhances neuronal survival after oxygen and glucose deprivation by increasing SIRT1. *European Journal of Pharmacology*.

[23] Li WW, Gao XM, Wang XM, Guo H, Zhang BL (2011). Icariin inhibits hydrogen peroxide-induced toxicity through inhibition of phosphorylation of JNK/p38 MAPK and p53 activity. *Mutation Research*.

[24] Liu B, Zhang H, Xu C (2011). Neuroprotective effects of icariin on corticosterone-induced apoptosis in primary cultured rat hippocampal neurons. *Brain Research*.

[25] Horiuchi H, Ogata T, Morino T, Chuai M, Yamamoto H (2003). Continuous intrathecal infusion of SB203580, a selective inhibitor of p38 mitogen-activated protein kinase, reduces the damage of hind-limb function after thoracic spinal cord injury in rat. *Neuroscience Research*.

[26] Xu Z, Wang BR, Wang X (2006). ERK1/2 and p38 mitogen-activated protein kinase mediate iNOS-induced spinal neuron degeneration after acute traumatic spinal cord injury. *Life Sciences*.

